# Venous thromboembolic events after bariatric surgery: Protocol for a systematic review and meta-analysis

**DOI:** 10.1016/j.isjp.2020.06.001

**Published:** 2020-06-13

**Authors:** Walid El Ansari, Brijesh Sathian, Ayman El-Menyar

**Affiliations:** a Department of Surgery, Hamad Medical Corporation, Doha 3050, Qatar; b College of Medicine, Qatar University, Doha 2713, Qatar; c Schools of Health and Education, University of Skovde, 541 28 Skövde, Sweden; d Department of Surgery, Trauma and Vascular Surgery, Clinical Research, Hamad General Hospital, Doha 3050, Qatar; e Clinical Medicine, Weill Cornell Medical School, Doha 24144, Qatar

**Keywords:** Systematic review, Metanalysis, Obesity, Morbid obesity, Bariatric surgery, Embolism and thrombosis, Prevention and control, Venous thromboembolism

## Abstract

**Introduction:**

Considerably large numbers of bariatric surgery (BS) procedures are undertaken globally, and are projected to increase with the obesity epidemic. Venous thromboembolic events (VTE) comprise an important cause of postoperative morbidity and mortality after BS and an important issue with wide clinical and financial repercussions. Yet, a precise extent of the prevalence of VTE after BS for obesity and its mortality remains uncertain.

**Methods and analysis:**

In order to respond to this knowledge gap, we will conduct a systematic review and meta-analysis of the prevalence of and mortality associated with VTE after BS. This protocol outlines the methodology that will be used and the search strategies and eligibility criteria that will be utilized to identify and select studies, as well as the method by which data from the selected studies will be extracted for analysis. PubMed, Cochrane Central Register of Controlled Trials (CENTRAL), WHO International Clinical Trials Registry Platform, Cochrane Library, MEDLINE, Scopus, clinicaltrials.gov and Google scholar will be searched from 01 January 1990 through 10th April 2020, for original studies written in English that provided prevalence estimates of VTE after BS. Articles will also be searched for mortality estimates of VTE after BS. STROCSS (Strengthening the Reporting of Cohort Studies in Surgery) criteria will evaluate the methodological quality of the selected studies. The use of fixed effect or random effects model will be subject to the findings of the statistical tests for heterogeneity. Publication bias will be visually estimated by inspecting the funnel plots. Pooled estimates will be computed. Th current protocol conforms to the Preferred Reporting Items for Systematic reviews and Meta-Analysis (PRISMA) guidelines and has been submitted to the PROSPERO International Prospective Register of systematic reviews. No ethical clearance is required for this study. This systematic review and meta-analysis will be published in a peer-reviewed journal and presented at national and international conferences.

## Highlights

• Venous Thromboembolic Events after Bariatric Surgery.
• This protocol undertakes a systematic review and metanalysis of venous thromboembolic events after bariatric surgery.
• Methodology, searches, eligibility, and data extraction for analysis are described.
• Following the PRISMA guidelines, it describes the approach for pooled estimates.
• Findings will have key clinical consequences for patient care.

## 1 Introduction

Despite improvements in bariatric surgery (BS), it remains a technically difficult operation performed on high-risk patients [1]. As the volume of BS increases globally, BS remains with risks and potential complications, of which venous thromboembolic events (VTE) comprise a significant cause of postoperative morbidity and mortality [2], [3]. Venous thromboembolic events comprise a range of incidents encompassing deep vein thrombosis (DVT) (including upper extremity DVT), pulmonary embolism (PE), and portomesenteric venous thrombosis (PMVT).

Bariatric surgery patients seem at a two-fold sets of risks for VTE: the risks due to obesity, and the risks associated with a demanding surgery. In terms of the former, obesity decreases mobility and leads to hypertension, diabetes, and obstructive sleep apnea among others, all of which are associated with VTE [4], [5], [6]; and, there is often venous stasis and chronic inflammation [7], and a general hypercoagulable state [8]. As for the latter, the longer operative time of surgery in obesity can increase the VTE risk; the creation of two separate anastomoses in some BS adds possibilities for endothelial vessel damage and VTE formation [9], [10], [11], [12]; and concomitant conditions (e.g. hernia repair) and complications (e.g. anastomotic leaks) [13] lead to extended immobility, hence increasing the VTE risk. Moreover, major bleeding that requires blood transfusion might also considerably increase the VTE risk [14]; and the optimal strategy for the prevention of VTE in the BS setting remains uncertain [15]. Indeed, obese patients undergoing bariatric procedures have an increased risk for VTE that surpasses the risk due to the surgery alone [16].

Venous thromboembolic events have considerable clinical significance. In primary BS, VTE were the third complication (0.3%, more common than leak 0.2%), and had the greatest effect on readmission and mortality rates [17]. PE is a leading cause of death post-BS, and a common finding at autopsy [18], [19], [20], [21]. The 30-day post-discharge VTE incidence was 0.29%, and among those having post-discharge VTE, mortality increased 28-fold (2.6% vs 0.09%; P < 0.001) [22]. Likewise, the incidence of fatal PE is uncertain. PE was found in 8 of 10 deceased patients post-BS; in 2 cases only, there was clinical suspicion of PE [21]. Post-BS PE has high mortality rate [18], particularly post-discharge mortality [19], [23], where autopsy found PE as leading cause of death in 20.7% deceased patients after BS [24]. Portomesenteric thrombosis mortality rate is 1.61% [25].

### 1.1 Rationale

On the one hand, based on individual studies, the literature suggests wide inconsistencies in the frequency of VTE. Reported rates of VTE, including deep vein thrombosis (DVT) and pulmonary embolism (PE) after BS range between 0.3% and 2.2%, with rates of PE being about 1%, despite the use of strategies to prevent these complications [26], [27], [28], [29], [30]. Others found an incidence of symptomatic DVT and PE post-BS of 0%–5.4% and 0%–6.4% respectively [31], [32], [33], [34].

On the other hand, published systematic reviews seem to have focused on various pharmacologic and mechanical prevention strategies of VTE in patients undergoing BS [35], [36], [37], [38], trends in DVT prophylaxis [39], or the safety of laparoscopic vs open BS [40]. Notably, one systematic review and meta-analysis examined the early major complications after BS [41]. However, this study [41] examined only early complications (≤ 30) days after surgery, when ≈80% of VTE appear after discharge [42], [43], and cumulative incidence increases with time [27], [42]; assessed only PE as a thromboembolic event (as opposed to the boarder range of VTE); focused only on publications originating from the USA (omitting reports from other parts of the world); and, evaluated the period 2003–2014. Another systematic review evaluated obesity as a risk factor for VTE in medical and bariatric patients, but is quite outdated (appraised literature published between 1976 and 2006) [16].

Despite such inconsistencies in reported prevalence of VTE and its associated mortality based on individual studies, there seems to be very little compilation and amalgamation of the available evidence in order to holistically understand the risk of VTE post-BS. For instance, there does not appear to be a recently published systematic review quantifying the prevalence of and mortality associated with VTE after BS. Moreover, there is no published meta-analysis of pooled findings from relevant studies across the globe in order to analyze, synthesize and appraise, with more precision, the risk of VTE and its associated mortality after BS. This is rather surprising, given that understanding the 'real' risk of VTE after BS appears to be critical to all subsequent actions of prevention policies and prophylaxis strategies aimed to reduce this serious and potentially life-threatening complication.

The gap in knowledge highlighted above, as well as the seriousness of VTE as complications and their clinical implications inspired the current systematic review and meta-analysis. The aim of the current protocol is to evaluate the risk of VTE and its associated mortality among patients who had undergone BS. We will undertake a systematic review and meta-analysis to assess the prevalence of VTE after BS for obesity regardless of the used regimen/s of prophylaxis. The systematic review and meta-analysis will also appraise the mortality associated with such prevalence of VTE. The protocol will address the question: “What is the prevalence of and mortality associated with VTE among patients who had undertaken BS?”.

## 2 Objectives

This protocol aims to evaluate the risk of VTE, portomesenteric vein thrombosis and associated mortality among bariatric surgery patients.

## 3 Methods

This systematic review and meta-analysis protocol was submitted to the PROSPERO International Prospective Register of systematic reviews (www.crd.york.ac.uk/PROSPERO) (ID: 184529, registration pending). The protocol is also registered with researchregistry (researchregistry5667, https://www.researchregistry.com/browse-the-registry#home/). The protocol will be reported in line with the Preferred Reporting Items for Systematic reviews and Meta-Analyses Protocol (PRISMA-P) Statement [44], [45].

### 3.1 Eligibility criteria

This protocol will identify studies on VTE after BS published from 01 January 1990 through 10th April 2020, in all settings. All studies meeting the eligibility criteria will be selected for extended review and synthesis. If more than one publication describes the same study, the one that provides the most data will be included in the meta-analysis. There will be no limitations on the type of BS conducted in the eligibility criteria, thus taking advantage of the range of research that addresses VTE after BS. The inclusion/exclusion criteria will be premised on whether a study provided detailed information on the association between VTE as a post-operative outcome among patients who had undertaken BS.

#### 3.1.1 Inclusion criteria and study selection

**Fig. 1:**
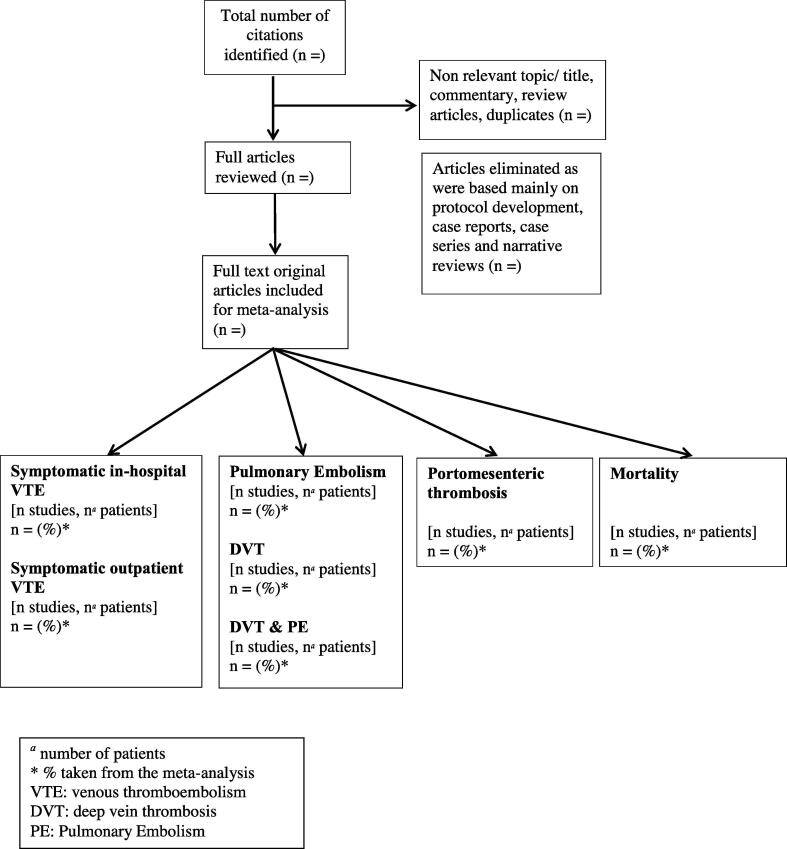
Flow diagram of study selection process for the systematic review.

Study design: (1) Original studies. Fig. 1 shows the flow diagram to be employed for the study selection process for the systematic review.

Language: (2) Published articles in English language.

Time Period: (3) Original studies published from 01 January 1990 through 10th April 2020.

Interventions: (4) Published articles that assessed “Venous Thromboembolism” and “bariatric surgery”.

Participants: (5) Published articles enrolling patients of any age, gender, and ethnicity in any country.

#### 3.1.2 Exclusion criteria

(1) Articles other than original studies such as commentaries, letters to the editor, reviews, conference proceedings, opinion papers, case reports.
(2) Studies that did not include outcomes or prevalences.

### 3.2 Information sources

This review will utilize a search strategy to detect and attain published and unpublished studies using bibliographic databases and grey literature. The search strategy emerged from examining available reviews of “Venous Thromboembolism” and “bariatric surgery”, in order to ascertain the appropriate bibliographic databases and search terms. Additional search terms will be included after consultation with an information specialist (librarian) at our institution experienced in systematic reviews.

### 3.3 Literature searches

A systematic review will be conducted out using PubMed, Cochrane Central Register of Controlled Trials (CENTRAL), WHO International Clinical Trials Registry Platform, Cochrane Library, MEDLINE, Scopus, clinicaltrials.gov and Google scholar electronic databases. We will use the keywords “bariatric surgery” “VTE” “DVT” “PE” “PORTOMESENTERIC” [in Title/Abstract]. The medical subject headings (MeSH) terms that will be used are bariatric surgery [All Fields] AND (“venous thromboembolism”[MeSH Terms]. Additional searches will be performed employing the reference lists of studies and review articles for a selection of relevant articles. The references of all included articles or relevant reviews will be cross-checked in order to ensure that no relevant items that could contribute to this proposed systematic review and meta-analysis have been missed.

### 3.4 Finding research evidence: three-step search strategy

This protocol will thoroughly search for prevalence and outcomes of venous Thromboembolism after BS. In order to accomplish this assignment, the protocol will employ a three-step search strategy combining academic bibliographies and internet searches:

Step 1: studies will be identified applying the predefined search strategy and bibliographic databases (see above) to systematically locate studies published from 01 January 1990 through 10th April 2020 that reported the prevelances and outcomes VTE after BS.

Step 2: screening will be performed on the reference lists of the retrieved articles, dissertations, and other studies that report on the prevelances and outcomes VTE after BS in obese patients.

Step 3: searching will be done of web-based platforms for studies using the above keywords in specialized journals, there will also be Google search for grey literature, as well as exhausting other global libraries for scientific literature.

### 3.5 Study records

#### 3.5.1 Data management

Citations retrieved from the bibliographic databases will be imported into Endnote in order to manage and remove any duplicates. Studies retrieved from reference lists of retrieved articles and Google search will be entered into an Excel spreadsheet for detection and deletion of duplicates and screening.

#### 3.5.2 Data extraction

The titles of the research articles attained from the initial database searches will be screened and applicable papers will be selected. Then the abstracts and full texts will be reviewed in keeping with the inclusion criteria for final selection. Three members of the research team will individually review the studies based on the exclusion and inclusion criteria. First, titles of the studies ascertained from the search will be appraised for possible inclusion. Titles approved by the authors will proceed to abstract screening. If three researchers decline a study at this point, it will be excluded from the review. Then, full text articles will be screened for eligibility. Only studies approved by the three researchers will be included. Concurrence between the researchers on the quality of the articles will be computed. All disagreements will be settled by consensus among the researchers. The reasons for exclusion will be observed.

#### 3.5.3 Data items to be extracted

We will extract several main categories of data: 1) (Author/s) names; (2) Year of publication; (3) Bariatric procedure undertaken; (4) Study design; (5) Sample or type of sampling; (6) Period of data collection; (7) Country where research was undertaken; (8) Number of patients in the study (sample size); (9) Sex of the patients enrolled in the study; (10) Mean age of patients enrolled in the study; (11) Number of patients; (12) Prevalence of venous thromboembolism; (13) Mortality; and, (14) Whether STROCSS (Strengthening the Reporting of Cohort Studies in Surgery) [46] appraisal was undertaken for the given study.

## 4 Outcomes

### 4.1 Primary outcomes

This protocol has one primary outcome, which will contribute to prevalence of venous thromboembolism after bariatric surgery.

### 4.2 Secondary outcomes

Where available, secondary outcomes for this review will include mortality after venous thromboembolism; and prevalence of venous thromboembolism after open and after laparoscopic bariatric surgery.

## 5 Assessment of methodological quality

An appraisal of risk of bias will be included into the analysis. We will scrutinize the quality of the studies that will be included in the meta-analysis, in order to gauge the strength of the body of evidence on the estimates. Methodological quality of the selected studies will be weighed using the STROCSS criteria from the checklist. The STROCSS criteria are relevant in the evaluation of the methodological quality of studies in surgery. This appraisal will stick to the same practice of the data collection process where disagreements will be settled by debate between the reviewers or with a third reviewer. A systematic review is premised on the secondary research of the published literature; hence, the quality of the included studies defines the quality level and reliability of the final findings.

## 6 Data analysis and synthesis

Prevalences will be calculated for categorical variables. The decision to choose either fixed effect or random effects model will depend on the findings of the statistical tests for heterogeneity. Data heterogeneity will be appraised using the Cochrane Q homogeneity test (significance set at p < 0.10). Where studies are statistically homogeneous, fixed effect model will be selected. A random effects model will be utilized where studies are statistically heterogeneous. The Higgin’s I^2^ test is the ratio of true heterogeneity to the total variation in observed effects [47]. A rough guide to interpretation of I^2^ test is 0–25%: might not be important; 25–50%: may represent moderate heterogeneity; 50–75%: may represent substantial heterogeneity; and > 75%: considerable heterogeneity. Publication bias will be visually estimated by appraising the funnel plots. Pooled estimates will be computed using R 3.5.1 software. The quality of this systematic review is foreseen to be high, having a high level of compliance with AMSTAR2 [48].

## 7 Ethics and dissemination plans

This systematic review and metanalysis will analyse and synthesize data from existing published and unpublished studies that are available in the public domain, hence ethics approval is not required. The findings from this protocol will be submitted to and published in peer-reviewed journals, presented at conferences and shared with relevant centres and institutions. We intend to update the review over time as seen appropriate.

## Funding

The research received no specific grant from any funding agency in the public, commercial or non-for-profit sectors. The authors thank Qatar National Library for funding the open access charges for the publication of this manuscript.

## Declaration of Competing Interest

The authors have no competing interests to declare.

## Registration of research studies

The protocol is registered with researchregistry. The unique identifying (UIN) number is: researchregistry5667. https://www.researchregistry.com/browse-the-registry#home/.
